# Prevalence of Cognitive Impairment before Prostate Cancer Treatment

**DOI:** 10.3390/cancers14051355

**Published:** 2022-03-07

**Authors:** Natália Araújo, Adriana Costa, Catarina Lopes, Luisa Lopes-Conceição, Augusto Ferreira, Filipa Carneiro, Jorge Oliveira, Samantha Morais, Luís Pacheco-Figueiredo, Luis Ruano, Vítor Tedim Cruz, Susana Pereira, Nuno Lunet

**Affiliations:** 1 EPIUnit-Instituto de Saúde Pública, Universidade do Porto, Rua das Taipas 135, 4050-600 Porto, Portugal; natalia.araujo@ispup.up.pt (N.A.); adriana.costa@ispup.up.pt (A.C.); catarina.lopes@ispup.up.pt (C.L.); luisa.conceicao@ispup.up.pt (L.L.-C.); samantha.morais@ispup.up.pt (S.M.); luis.ruano@ispup.up.pt (L.R.); vitor.tedimcruz@ulsm.min-saude.pt (V.T.C.); susana.pereira@ipoporto.min-saude.pt (S.P.); 2 Laboratório para a Investigação Integrativa e Translacional em Saúde Populacional (ITR), Rua das Taipas 135, 4050-600 Porto, Portugal; 3 Instituto Português de Oncologia do Porto, Rua Dr. António Bernardino de Almeida, 4200-072 Porto, Portugal; augusto.carmo.ferreira@ipoporto.min-saude.pt (A.F.); ana.carneiro@ipoporto.min-saude.pt (F.C.); jorge.oliveira@cuf.pt (J.O.); 4 Instituto de Investigação em Ciências da Vida e Saúde, Escola de Medicina da Universidade do Minho, 4710-057 Braga, Portugal; lfigueiredo@med.uminho.pt; 5 Departamento de Ciências da Saúde Pública e Forenses e Educação Médica, Faculdade de Medicina da Universidade do Porto, Alameda Professor Hernâni Monteiro, 4200-319 Porto, Portugal

**Keywords:** prostate cancer, prevalence study, cognitive dysfunction/epidemiology, neuropsychological test

## Abstract

**Simple Summary:**

The experience of a cancer diagnosis, cancer treatments, and the tumor itself may have impacts on cognitive performance. Patients with prostate cancer are a large population who may be more vulnerable to cognitive deterioration due to older age. Understanding the occurrence of cognitive impairment among these patients is essential for developing strategies aiming to slow down the progression of cognitive deterioration, to increase patients’ autonomy and quality of life, and to decrease the probability of non-compliance with treatment. This study describes the prevalence of cognitive impairment in patients recently diagnosed with prostate cancer and proposed for any type of cancer treatment. No significant differences were observed between the frequency of cognitive impairment in these patients before treatment and in the general population, suggesting that the impact of prostate cancer on cognitive performance could be negligible in the short term, contrary to what other studies have reported in other types of cancer.

**Abstract:**

Cognitive impairment is common among patients with different types of cancer, even before cancer treatment, but no data were reported among patients with prostate cancer (PCa), who may be at high risk due to advanced age. This study aims to estimate the prevalence of cognitive impairment before PCa treatment. Between February 2018 and April 2021, the NEON-PC cohort recruited 605 patients with PCa proposed for treatment at the Portuguese Institute of Oncology of Porto. The Montreal Cognitive Assessment (MoCA) was used to assess cognitive performance. Participants with a MoCA < 1.5 standard deviations (SD) of age- and education-specific normative values were considered to have probable cognitive impairment (PCI) and were referred for a comprehensive neuropsychological assessment. Data from the population-based cohort EPIPorto (*n* = 351 men aged ≥40 years, evaluated in 2013–2015) were used for comparison. The prevalence of PCI was 17.4% in EPIPorto and 14.7% in NEON-PC (age- and education-adjusted odds ratio: 0.82, 95%CI: 0.58,1.18). Neuropsychological assessment was performed in 63 patients with PCa: 54.0% had cognitive impairment. These results suggest that the impact of PCa on cognitive performance could be negligible in the short term, contrary to what other studies have reported regarding other types of cancer.

## 1. Introduction

Cognitive impairment in cancer patients has been the focus of numerous studies over the few last decades, mostly regarding the effects of cancer treatments on this condition and, more recently, its occurrence before cancer therapy [1]. Cognitive impairment may have an important impact on the patients’ and their families’ lives, as well as on cancer treatments. Patients diagnosed with mild cognitive impairment (MCI) have reported diminished self-confidence, as well as fear of embarrassment and concerns about changing family roles due to cognitive impairment, and their family members have reported that these patients have an increased dependence on others [2]. Moreover, patients with MCI may present difficulties in medical decisions, namely in appreciating the consequences of treatment choice, providing rational reasons for treatment choice, and understanding the treatment situation and choices [3], as well as reduced adherence to treatment due to memory and executive functions impairments [4]. Cognitive complaints in cancer patients may interfere with occupational reintegration and working performance, which has an important impact on patients’ quality of life, mental health and finances, and, at the societal level, on work capacity and productivity [5].

Pathophysiological processes induced by cancer, the experience of a cancer diagnosis, or cancer treatment may negatively impact cognitive performance, and cognitive impairment has been often reported among these patients [6,7]. However, cancer is not a single disease, and even among tumors with the same typology, there is a large heterogeneity regarding cancer-related symptoms, including those related with the impairment of the patients’ cognitive status [8,9].

In some longitudinal studies aiming to assess the impact of chemotherapy on cognitive performance, cognitive impairment was reported to be frequent before treatment initiation: in 11% [10] to 35% [11] of patients with breast cancer, in 46% of patients with testicular cancer [12], and in 45% of patients with colorectal cancer [13]. In patients with small-cell lung cancer, 70% had impairment in verbal memory, up to 30% in frontal lobe executive functions, and one-third in motor coordination, which was attributed to paraneoplastic syndrome by the authors; the latter is rare in most cancers, but may affect 10% of patients with small-cell lung cancer [14]. Alterations in cytokine serum levels observed in patients with acute myelogenous leukemia or myelodysplastic syndrome [15] and in women with breast cancer [16] have also been associated with impairment in certain cognitive domains. Post-traumatic stress syndrome related to cancer diagnosis observed in women with breast cancer may also explain impaired performance in cognitive tests [17].

Prostate cancer is the most prevalent neoplasm among men [18] due to its high incidence rates and overall good prognosis. The cognitive performance of patients with this cancer has been studied in the context of the association of androgen deprivation therapy (ADT) with cognitive decline [19] and dementia [20], which could be explained by testosterone castration levels induced by ADT and the loss of the neuroprotective effect of this hormone [21]. Patients frequently present advanced age at diagnosis (median age of 67 years [22]), which is a known risk factor for cognitive impairment [23]. Moreover, insulin resistance has been associated with the increased risk of prostate cancer [24] and of cognitive impairment [25]. Therefore, these risk factors and the possible effect of cancer on cognitive function, as suggested in other populations of cancer patients, highlight the importance of knowing the prevalence of cognitive impairment among patients with prostate cancer even before cancer treatment. It is important to inform patients and their family, clinicians, and health institutions about the burden of prostate cancer in the initial phase of the disease, as well as for evidence-based counselling and provision of care.

Although the prevalence of cognitive impairment was reported to vary between 10% and 69%, these values refer to the percentage of men who presented cognitive decline, which is a measure of cognitive variation from before ADT to months after the baseline evaluation, and not to the impairment of cognitive function with regard to what would be expected to be normal cognitive functioning according to age and education [26]. Indeed, only one study reported the prevalence of cognitive impairment before ADT based on scores below specific normative cut-off values on cognitive tests [27], but patients proposed for non-hormonal treatment were not included in the study, and 85% of participants had biochemical relapse. Another study reported the percentage of cognitive impairment before ADT, but patients performing low in a cognitive screening instrument were not included, and prostate cancer had been diagnosed years before the study [28].

Therefore, this study aims to estimate the prevalence of cognitive impairment in patients recently diagnosed with prostate cancer before cancer treatment. A global measure of cognitive performance is compared between patients with prostate cancer and men in the general population. Among patients with prostate cancer, the prevalence of impairment in each cognitive domain and in the overall cognitive performance is described. Cancer stage, which reflects the extent and aggressiveness of the cancer, and treatment proposal, which reflects cancer stage, but also the patients’ preferences for therapy, fears of treatment, and their consequences, are considered as potential contributors to cognitive impairment.

## 2. Materials and Methods

This study is based on cross-sectional evaluations of the NEON-PC cohort of patients with prostate cancer and the EPIPorto cohort of the general population.

### 2.1. NEON-PC Cohort

This prospective cohort study took place at the Portuguese Institute of Oncology of Porto (IPO-Porto), which is one of the largest cancer hospitals in Portugal, mainly providing care to patients in the northern region after a referral from the family doctor or according to public hospital collaboration protocols.

The study protocol was previously described in detail [29]. Briefly, between March 2018 and April 2021, patients recently diagnosed with prostate cancer and expected to be treated at IPO-Porto were considered eligible. Patients with less than one year of education, those who were not Portuguese native speakers, those with a history of chemotherapy, radiotherapy, or androgen deprivation therapy treatments, or with previously diagnosed neurologic or psychiatric conditions impairing cognitive performance were excluded. Due to the COVID-19 pandemic, field activities at IPO-Porto were suspended from 9 March to 30 June 2020. A total of 605 patients agreed to participate, 98 refused, and in 32 cases, the evaluation could not be performed before treatments due to the COVID-19 pandemic.

#### 2.1.1. Evaluation of Participants’ Cognitive Performance

The Montreal Cognitive Assessment (MoCA) is a cognitive test designed to detect mild cognitive impairment, showing good sensitivity and specificity [30]. Version 7.1, which is validated in the Portuguese population, was used in the current study [31]. MoCA assesses executive functions, visuospatial ability, short-term memory, language, attention, concentration, working memory, and temporal and spatial orientation through 12 tasks. The overall score ranges from 0 to 30, with higher scores corresponding to better cognitive performance. Participants scoring below 1.5 standard deviations (SD) of age- and education-specific normative values [32] were considered to present probable cognitive impairment (PCI).

In the NEON-PC cohort, participants with PCI were invited to perform a comprehensive neuropsychological assessment with a trained neuropsychologist. The battery of tests assessed verbal and visual memory, working memory, information processing speed, executive functions, and language using tests validated in the Portuguese population: the Wechsler Memory Scale Third Edition [33], Wechsler Adult Intelligence Scale Third Edition [34], Trail Making Test [35], Stroop test [36], Phonemic Verbal Fluency [37], Clock drawing test [38], and the Token Test-short-form [39]. For each cognitive domain, the criterion used for the classification of cognitive impairment was based on the number of tests used to assess the cognitive domain and the number of scores below age-corrected norm cut-off values (below 1, 1.5, or 2 SD) [40], as described in detail in Table 1. Patients were classified as having cognitive impairment when at least one cognitive domain was impaired.

A total of 10 participants refused to perform this evaluation, 4 abandoned the study, and the evaluation could not be performed in 13 participants; as such, 65 patients completed the neuropsychological assessment. Those who underwent the neuropsychological assessment were not statistically different from those who did not in terms of age (*p* = 0.553), education (*p* = 0.164), and the treatment proposed to treat prostate cancer, either ADT +/− chemotherapy or other treatments (*p* = 0.745).

#### 2.1.2. Assessment of Anxiety and Depression and Clinical Information in the NEON-PC Cohort

Patients with prostate cancer completed the Hospital Anxiety and Depression Scale [41,42]. Anxiety and depression sub-scores equal to or higher than 11 out of a possible 21 were considered indicative of clinically significant anxiety or depression symptoms. Information on tumor size (T), invasion of lymph node (N) and metastases (M), Gleason score, and prostate-specific antigen (PSA) were retrieved from medical files and used to assign prostate cancer prognostic stage groups according to the American Joint Committee on Cancer TNM staging system, eighth edition [43]. Gleason scores were grouped into Gleason grades according to the International Society of Urological Pathology [44]. Data on co-morbidities were obtained using self-reports and completed with information from medical records. Participants were also asked about the regular consumption of medication, and drugs were classified according to the Anatomical Therapeutic Chemical Classification System [45].

### 2.2. EPIPorto Cohort

EPIPorto is a population-based closed cohort assembled between 1999 and 2003 in the city of Porto (≈400,000 inhabitants), representative of dwellers aged 18 years or older (*n* = 2485). Random digit dialing of landline telephones was used to select households, and a permanent resident aged at least 18 years was selected within each household, by simple random sampling, with a participation rate of 70% [46]. A total of 354 male participants aged 40 or older were tested with MoCA in the 2013–2015 re-evaluation of the cohort [47]. In accordance with the exclusion criteria used in the NEON-PC cohort, three participants who presented with Parkinson’s or Alzheimer’s diseases were excluded.

Participants of the EPIPorto cohort were younger (mean, standard deviation: 63.8, 11.1 vs. 68.1, 7.3, *p* < 0.001) and with a higher education level than participants of the NEON-PC cohort (P25, median, P75: 56, 64, 71 vs. 63, 68, 74, *p* < 0.001 and 5, 9, 15 vs. 4, 4, 9, *p* < 0.001, respectively).

### 2.3. Data Analysis

A total of 605 patients with prostate cancer (NEON-PC) and 351 men from the general population (EPIPorto) were considered for analysis.

Sample characteristics are presented as counts and proportions for categorical variables, and median, 25th and 75th percentiles for quantitative variables. Multivariate logistic regression was used to estimate the age- and education-adjusted odds ratio (aOR) of the association between belonging to the NEON-PC cohort vs. to the EPIPorto cohort with the presence of PCI, and the association between socio-demographic and clinical variables with PCI, among patients with prostate cancer.

All analyses were performed using STATA v.15 (StataCorp. 2017. Stata Statistical Software: Release 15. StataCorp LLC, College Station, TX, USA). All tests were two sided, and a *p* < 0.05 was considered significant.

### 2.4. Ethics Approval

Ethics approval was obtained from the Ethics Committee of the IPO-Porto for the NEON-PC cohort (CES 089/017) and from the Ethics Committee of the Hospital de São João for the EPIPorto cohort (CE HSJ n°65-20/10/95). The study was carried out according to the Helsinki Declaration, and all participants completed the informed written consent form.

## 3. Results

In the NEON-PC cohort, 89 participants (14.7%) presented PCI, whereas in the EPIPorto cohort 61 (17.4%) did so, corresponding to an age- and education-adjusted odds ratio of 0.82 (95%CI: 0.58, 1.18).

Table 2 presents the characteristics of participants with prostate cancer. Most had prognostic cancer stage group I/II (66.2%), and 13.4% of participants had prognostic cancer stage IV (positive lymph nodes and/or metastases), of which, some (N1M0) were eligible for radiotherapy with ADT. Only 4.0% had a proposal of ADT with docetaxel. Patients with a proposal of ADT +/− chemotherapy were older (mean, standard deviation, in years: 70.7, 7.0 vs. 67.8, 7.3, *p* = 0.005).

**Figure 1:**
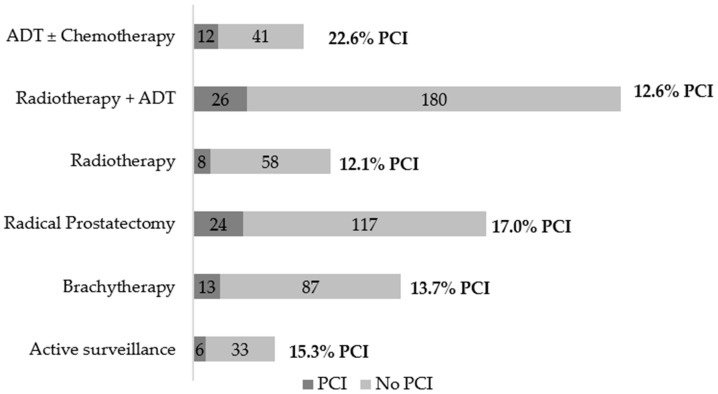
Participants with probable cognitive impairment (PCI) according to the proposal of treatment.

Figure 1 presents the distribution of PCI among patients with prostate cancer proposed for different treatments. PCI was more frequent (22.6%) among those proposed for ADT alone or with chemotherapy, and less frequent (12.1%) among those proposed for radiotherapy (external beam radiation with no hormonal treatment).

Table 3 presents the associations between sociodemographic and clinical variables with PCI among patients with prostate cancer. Educational level 5–9 years (vs. <4 years) was associated with higher odds of PCI (age-adjusted OR, 95%CI: 2.49, 1.45–4.28). Patients with co-morbidities were more likely to present PCI, although only the association of having a diagnosis of a lung disease (asthma, chronic obstructive pulmonary disease, chronic bronchitis, surgery for lung cancer, pulmonary emphysema, and pneumonia) was nearly significant (age- and education-adjusted OR, 95%CI: 1.87, 0.97–3.60). Participants presenting with depression were more likely to have PCI, although this was a non-statistically significant result (age- and education-adjusted OR: 2.51, 0.93–6.76). Although the association after adjustment for age and education did not reach a statistical significance (OR, 95%CI: 1.91, 0.95–3.87), participants proposed for ADT alone or with chemotherapy were more likely to present PCI.

Considering patients with prostate cancer and PCI who completed the neuropsychological assessment, 38.1% had normal cognitive function, 7.9% had mild deficits (one or more cognitive scores below 1.0 SD of age-corrected norms but without fulfilling the criterion for cognitive impairment), and 54.0% had cognitive impairment.

Table 4 presents the number of participants with impairment in each cognitive domain. Executive function was the most affected domain, being impaired in 47.6% of the participants who performed the neuropsychological assessment. One participant had impairment in executive functions, while in the other cognitive domains, his scores were within the normal range. All of the remaining participants had at least one additional cognitive domain with a score below the normal range, either showing dysfunction or impairment.

## 4. Discussion

The prevalence of PCI was similar in patients with prostate cancer and in the general population. Patients proposed for ADT alone or with chemotherapy presented PCI more frequently than patients proposed for other treatments. Cognitive impairment was confirmed by neuropsychological testing in just over half the patients with PCI, and executive function was the most frequently impaired domain.

The prevalence of cognitive impairment in the present study, detected with MoCA and with a neuropsychological test battery, was much lower than in previous studies performed in patients with other cancers [10,11,12,13,14,48]. Prostate cancer, which is an indolent and localized disease in many men, may not induce the same pattern of systemic pathophysiologic alterations as other cancers, which might be a cause of cognitive impairment.

Moreover, there is no gold standard for measuring cognitive function, and the different methods used to evaluate cognitive performance and define cognitive impairment may also explain the heterogeneous results. Indeed, the cognitive tests and the cognitive domains they assess, the number of tests, and the criterion used to classify cognitive impairment vary substantially across studies [1]. Using the criterion for the classification for cognitive impairment based on presenting at least one score below the cut-off of 2 SD of the norms is associated with a 5% probability of misclassification due to chance only if one out of two administered tests is below the cut-off. This probability increases with the number of tests administered, and if one out of nine tests is below the cut-off, then the probability of misclassification is more than 20%. Likewise, using the criterion of presenting at least two scores below 1.5 SD of the norm is associated with a 5% probability of misclassification if two out of six scores are below the cut-off and more than 20% if two out of twelve scores are below the cut-off [40]. Therefore, misclassification due to chance could explain the high values for cognitive impairment reported in patients with cancer in other studies. We classified cognitive impairment in each cognitive domain, considering three factors: the number of tests administered, the cut-off based on age-corrected norms (1, 1.5 or 2 SD), and depending on the two others, the number of tests with a score below the cut-off, to not exceed by 5% the probability of misclassification [40].

Among men with prostate cancer, there is only one previous study reporting the prevalence of cognitive impairment before ADT [27]. In addition to the criteria used to define cognitive impairment, the particular characteristics of the sample could explain the high value of 45%. Indeed, 15% of the patients had asymptomatic metastatic disease, and 85% of the patients had biochemical relapse [27], that is, most of the patients were previously treated for prostate cancer, and frequent sequelae of previous treatment, such as anemia [49] and depression [50], may have contributed to an increased prevalence of cognitive impairment [51,52] compared to patients recently diagnosed with prostate cancer. In this study, participants were classified with cognitive impairment when presenting two low scores, and these were most frequently observed in tasks assessing memory and executive functions, which is in accordance with our findings.

Older age is considered to increase the likelihood for cognitive impairment, while higher education is associated with decreased risk [23]. However, in the NEON-PC cohort, PCI was more frequent in participants with five to nine years of school attainment than in less educated individuals. This result deserves further confirmation in other studies. Several sociodemographic, lifestyle, and clinical characteristics of the patients were analyzed, but none contributed to explain this association with PCI.

Depression may impair performance in cognitive tests, particularly in an elderly population [52,53]. Among patients with prostate cancer, the association between depression and PCI was not statistically significant. However, the prevalence of depression was low, which contributes to limited statistical power. Previous studies conducted among patients with prostate cancer before ADT did not report on the effect of depression on cognitive impairment [27,28].

The association of lung disease with PCI is supported by previous results showing that 32% of patients with chronic obstructive pulmonary disease has cognitive impairment [54,55].

Our results show PCI may be more frequent in patients with advanced disease proposed for ADT. This may contribute to explaining the conflicting results regarding cognitive decline from studies that only included patients who would receive radiotherapy with ADT and showed no effect of ADT on cognitive performance over time [56] and others that did not include these patients but only those to be treated with androgen ablation and reported a negative effect of ADT on cognitive tests [57].

### Strengths and Limitations

This is the first study to report the prevalence of cognitive impairment in a large cohort of patients with prostate cancer, including patients proposed for several different treatments.

We used data from the EPIPorto population-based cohort for comparison, which allowed us to consider the prevalence of cognitive impairment in patients with prostate cancer as similar to that observed in the general population. The control group is of increased importance when the definition of the outcome differs from study to study, making the appreciation of the findings difficult. Indeed, two studies reported similar values of the prevalence of cognitive impairment in patients with prostate cancer, 45% and 41%, which may be considered worrying values, but in the latter, the age- and education-matched control group also presented a prevalence of 44% for cognitive impairment.

EPIPorto was a representative sample of the population of the city of Porto in 1999–2003 and suffered from attrition since its assembling to the third evaluation in 2013–2015. It is more likely that the participants who abandoned the study had higher odds of cognitive impairment [58]. On the other hand, IPO-Porto mostly admits patients from the northern region, and urban Portuguese areas have a lower prevalence of cognitive impairment than rural areas [59]. Thus, the prevalence of PCI in the EPIPorto cohort may be lower than it would be in a newly assembled cohort representative of the Portuguese northern region, and it is not likely that PCI would be more frequent in patients with prostate cancer than in the general population.

## 5. Conclusions

The likelihood of PCI was similar among patients with recently diagnosed prostate cancer before treatment and in the general population. This finding is rather reassuring for patients and their family who are the most affected by the consequences of cancer and for clinicians and health institutions who have to plan clinical protocols and resources to deliver the most adequate treatment to patients with prostate cancer. These results suggest that the impact of prostate cancer on cognitive performance could be negligible in the short term, contrary to what other studies have reported regarding other types of cancer.

## Figures and Tables

**Table 1:** Criteria used for the classification of cognitive impairment, considering the number of tests administered to assess each cognitive domain.

Cognitive Domain	Test	Criteria for Impairment
Verbal memory	WMS III—Logical memory I and II	2 scores < 1.5 SD or 1 score < 2 SD
Visual memory	WMS III—Visual reproduction I and II	2 scores < 1.5 SD or 1 score < 2 SD
Working memory	WMS III—Digit span	score < 2 SD
Processing speed	WAIS III—Digit-Symbol-Coding and Symbol search	at least 3 scores < 1 SD or 2 scores < 1.5 SD
	Trail Making Test, part A	
	Stroop test—word reading	
Executive functions	Stroop test (color naming and word color naming)	at least 3 scores < 1 SD or 2 scores < 1.5 SD
	Trail Making Test, part B and B-A	
	Phonemic Fluency—letters M, R and P	
	Phonemic Fluency—categories of animals	
	18-points Clock drawing test	
Language	Token Test—short-form	score < 2 SD

SD, standard deviation; WAIS III, Wechsler Adult Intelligence Scale Third Edition; WMS III, Wechsler Memory Scale Third Edition.

**Table 2:** Sociodemographic and clinical characteristics of the participants.

Characteristics	All	ADT +/− Chemotherapy	Other Tx	Comparisons
	mean (sd)	mean (sd)	mean (sd)	*p* value
Age (years)	68.1 (7.3)	70.7 (7.0)	67.8 (7.3)	0.005
Education (years)	7.3 (4.9)	6.6 (4.2)	7.4 (4.9)	0.259
	*N* (%)	*N* (%)	*N* (%)	*p* value
Co-morbidities ^a^				0.944
None	110 (18.2)	9 (17.0)	101 (18.3)	
1–2	358 (59.2)	31 (58.5)	327 (59.2)	
≥3	137 (22.6)	13 (24.5)	124 (22.5)	
Heart disease	99 (16.3)	10 (18.9)	89 (16.0)	0.590
Stroke	20 (3.3)	2 (3.8)	18 (3.2)	0.834
Diabetes	127 (20.9)	17 (32.1)	110 (19.8)	0.035
Lung disease	63 (10.3)	4 (7.5)	59 (10.6)	0.484
Psychiatric disorder	42 (6.9)	1 (1.9)	41 (7.4)	0.132
Neurologic disorder	19 (3.1)	0 (0.0)	19 (3.4)	0.172
Consumption of psycholeptics ^b^				0.326
Yes	57 (9.4)	3 (5.7)	54 (9.8)	
Consumption of psychoanaleptics ^b^				0.214
Yes	50 (8.3)	2 (3.8)	48 (8.7)	
Anxiety ^c^				0.406
Yes	53 (9.2)	3 (6.0)	50 (9.6)	
Depression ^c^				0.405
Yes	22 (3.8)	3 (6.0)	19 (3.6)	
Prognostic cancer stage group ^d^				<0.001
I	45 (7.4)	0 (0.0)	45 (8.2)	
II	356 (58.8)	0 (0.0)	356 (64.5)	
II/III	7 (1.2)	0 (0.0)	7 (1.3)	
III	116 (19.2)	3 (5.7)	113 (20.5)	
IV	81 (13.4)	50 (94.3)	31 (5.6)	
Proposal of treatment				<0.001
Active surveillance	39 (6.4)	0 (0.0)	39 (7.1)	
Brachytherapy	100 (16.5)	0 (0.0)	100 (18.1)	
Radical prostatectomy	141 (23.3)	0 (0.0)	141 (25.5)	
Radiotherapy	66 (10.9)	0 (0.0)	66 (12.0)	
Radiotherapy + ADT	206 (34.0)	0 (0.0)	206 (37.3)	
ADT	29 (4.8)	29 (54.7)	0 (0.0)	
ADT + chemotherapy	24 (4.0)	24 (45.3)	0 (0.0)	

ADT, androgen deprivation therapy; sd, standard deviation; Tx, treatments for prostate cancer other than ADT +/− chemotherapy. ^a^ Co-morbidities reported by patients and retrieved from medical files. ^b^ Medication was reported by patients and retrieved from medical files. Drugs were classified according to the Anatomical Therapeutic Chemical Classification System [36]. ^c^ Anxiety/depression was considered when the Hospital Anxiety and Depression Scale (HADS) sub-score for anxiety/depression was ≥11. ^d^ Prostate cancer prognostic stage groups were assigned according to the American Joint Committee on Cancer TNM staging system, eighth edition [34].

**Table 3:** Adjusted odds ratio (OR) of the associations between sociodemographic characteristics, clinical characteristics of the tumor, and patient-reported outcomes—anxiety and depression—with probable cognitive impairment, among patients with prostate cancer.

Variable (Reference Category)	OR (95%CI)	Adjustment
Age (ref. <68 years)		
≥68 years	1.11 (0.71, 1.74)	
Education (ref. ≤4 years)		
5–9 years	2.49 (1.45, 4.28)	a
>9 years	1.23 (0.68, 2.22)	a
Cancer stage (ref. stage I)		
Stage II	1.21 (0.48, 3.04)	b
Stage III	0.83 (0.28, 2.43)	b
Stage IV	1.04 (0.35, 3.10)	b
Comorbidities (ref. none)		
1 or 2	1.47 (0.75, 2.90)	b
>2	1.73 (0.81, 3.69)	b
Previous diagnoses		
Heart disease vs. no heart disease	0.82 (0.43, 1.56)	b
Stroke vs. no stroke	2.25 (0.78, 6.47)	b
Diabetes vs. no diabetes	1.39 (0.82, 2.36)	b
Lung disease vs. no lung disease	1.87 (0.97, 3.60)	b
Psychiatric disorder vs. no psychiatric disorder	1.34 (0.59, 3.03)	b
Neurologic disorder vs. no neurologic disorder	1.42 (0.38, 5.26)	b
HADS classification		
Anxiety vs. no anxiety	1.54 (0.71, 3.35)	b
Depression vs. no depression	2.51 (0.93, 6.76)	b
Proposal of treatment		
ADT +/− chemotherapy vs. other treatments (AS, BqT, RP, RT, RT + ADT)	1.91 (0.95, 3.87)	b

ADT, androgen deprivation therapy; AS, active surveillance; BqT, brachytherapy; CI, confidence interval; HADS, Hospital Anxiety and Depression Scale; RP, radical prostatectomy; RT, radiotherapy. a, adjusted for age; b, adjusted for age and education

**Table 4:** Participants with prostate cancer who performed the neuropsychological assessment and presenting impairment in each cognitive domain.

Cognitive Domain	Participants with Normal Functioning ^a^ *n* (%)	Participants with Dysfunction ^b^ *n* (%)	Participants with Impairment ^c^ *n* (%)
Verbal memory	44 (69.8)	13 (20.0)	6 (9.5)
Visual memory	38 (60.3)	21 (33.3)	4 (6.4)
Working memory	53 (84.1)	10 (15.9)	0
Processing speed	40 (63.5)	19 (30.2)	4 (6.4)
Executive functions	24 (38.1)	9 (14.3)	30 (47.6)
Language	57 (90.5)	4 (6.4)	2 (3.2)

^a^ Normal functioning in each cognitive domain was considered when all scores were within the normal range (≥1 standard deviation (SD) below mean). ^b^ Dysfunction in each cognitive domain was considered when one or more scores were below the normal range (<1 SD) but the criteria for cognitive impairment were not fulfilled. ^c^ Cognitive impairment in each cognitive domain was considered according to the following criteria: 1 score < 2 SD, for working memory and language; at least 2 scores < 1.5 SD or 1 score < 2 SD, for verbal and visual memories; at least 3 scores < 1 SD or 2 scores < 1.5 SD, for processing speed and executive functions.

## Data Availability

The data presented in this study are available on request from the corresponding author. The data are not publicly available due to the fact that included patients do not specifically provide their consent for public sharing of their data and that anonymization is unlikely to be feasible, since the identification of patients treated in only one institution within a relatively short period may be possible when taking socio-demographic and clinical characteristics into account.
